# Evaluating the Role of Surgical Telementoring in the Acquisition of Surgical Skills in Laparoscopic Cholecystectomy: Protocol for a Pilot Randomized Controlled Trial

**DOI:** 10.2196/73159

**Published:** 2026-04-17

**Authors:** Khayam Butt, Laurent Olivier Trichet, Gunnar Hartvigsen, Ole Sjo, Bjørn von Gohren Edwin, Knut-Magne Augestad

**Affiliations:** 1 Department of Surgery, Nordland Hospital Trust, Parkveien 96, Bodø, 8005, Norway, 99560985; 2 Department of Research, Nordland Hospital Trust, Bodø, Norway; 3 Institute of Informatics, UiT The Arctic University of Norway, Tromsø, Norway; 4 Department of Hepatic, Gastrointestinal and Paediatric Surgery, Oslo University Hospital, Oslo, Norway; 5 The Intervention Center, Medical Faculty, Oslo University Hospital, Oslo, Norway; 6 Department of Surgery, Akershus University Hospital, Lillestrøm, Norway

**Keywords:** surgical telementoring, pilot randomized controlled trial, technical and nontechnical surgical skills, interprofessional education, competency-based education, videoconferencing, surgical education, laparoscopic cholecystectomy

## Abstract

**Background:**

Developing surgical skills among residents requires time and resources, often in conflict with the goals of efficiency and hospital economics in surgical practice. Optimizing resident training may prevent a decline in quality. Skilled surgeons are vital to effective and safe surgical performance, yet the training trajectory for surgical residents is often inefficient, particularly in the era of minimally invasive surgery. A framework that efficiently disseminates surgical skills is essential to cultivate skilled future surgeons within an acceptable time frame. Surgical telementoring (ST) aligns well with minimally invasive surgery because the mentee operating in the field shares the telementor’s perspective. Despite growing experience with telemedicine, data on educational outcomes remain limited.

**Objective:**

We aim to conduct a pilot randomized controlled trial (RCT). Data collection will inform a future full-scale RCT evaluating whether real-time ST, combined with structured postoperative coaching, is superior to traditional on-site mentoring in developing laparoscopic cholecystectomy skills among surgical residents.

**Methods:**

Eligible residents will be randomly assigned in a 1:1 ratio to either the intervention group (real-time ST and postoperative coaching) or the control group (traditional intraoperative hands-on teaching). This research adheres to the extended SPIRIT (Standard Protocol Items: Recommendations for Interventional Trials) statements for pilot RCTs and follows the intention-to-treat principle. Residents in both groups will perform 5 consecutive procedures under standardized intraoperative conditions. The primary outcomes are the Global Operative Assessment of Laparoscopic Skills (GOALS) score and the Non-Technical Skills for Surgeons (NOTSS). We hypothesize that the intervention group will show a 3- to 5-point improvement in the GOALS score compared with the control group. To achieve 0.8 statistical power, a *P* value <.05, and a 20% dropout rate in a definitive RCT, 12 residents per group are required. We present a pilot RCT with 5 residents per group. Challenges encountered during the trial will be reported, and amendments will be proposed to assess the need for a future definitive RCT.

**Results:**

The primary outcomes will be technical and nontechnical surgical skills, measured by the GOALS and NOTSS scores, respectively. Patient-reported experience measures and satisfaction scores of eligible mentees and mentors will be analyzed as secondary outcomes. The ST system configuration at the 2 inclusion centers was completed in December 2023. Ethics approval and the most recent update report to ClinicalTrials.gov were obtained on October 23, 2023, and December 8, 2024, respectively. Recruitment of residents to the study commenced in spring 2024. Recruitment is expected to be completed in May 2026, with data analysis in November 2026 and manuscript submission of results in January 2027.

**Conclusions:**

The SURGTEACH (Surgical Telementoring in Acquisition of Surgical Skills of Laparoscopic Cholecystectomy) trial is the first pilot RCT evaluating ST for educational outcomes. This research may provide high-quality evidence to improve surgical education, particularly in rural hospitals.

## Introduction

Developing surgical skills among residents is a time- and resource-consuming process [1]. Increasing demands for efficiency and hospital economics in surgical practice have contributed to a decrease in the number of surgeons per capita [2]. Concerning reports show increasingly limited access to the operating room (OR) during a surgical residents’ educational path [3]. Hence, efficient training trajectories for general surgeons are needed to ensure educational quality and efficiency [4].

Geographical challenges, including long distances and harsh weather conditions in Northern Norway, necessitate complex transportation arrangements to reach the hospital. A healthcare policy has been established that requires rurally located institutions to provide basic surgical care within an acceptable proximity to patients [5]. However, these institutions have limited access to surgical expertise, which may contribute to reduced quality of OR training for residents. The Norwegian National Medical Association has already taken this into account. This is one of the leading reasons for the reduced number of surgical procedures required to meet the Norwegian national requirements for achieving a specialty in general and colorectal surgery.

The centralization of surgical care, while improving outcomes for complex procedures at high-volume centers, has led to a decline in surgical services at smaller hospitals and greater challenges in recruiting and retaining consultant surgeons in rural areas.

This phenomenon is particularly pronounced in Scandinavian countries, where vast geographic distances amplify the impact of centralization policies. Norwegian rural hospitals increasingly rely on rotating surgeons and visiting consultants, creating discontinuity in surgical training programs and limiting residents’ exposure to consistent mentorship. A consequence of centralization is a lack of expert mentorship and a reduction in patient volume. Increased use of simulation training, surgical telementoring (ST), and information and communication technologies in many aspects of health care delivery are interventions being adopted to address these gaps [5-7]. These structural challenges underscore the need for innovative educational solutions that can deliver expert surgical guidance regardless of physical location.

Laparoscopic cholecystectomy (LC) is a technically challenging procedure that residents become familiar with early on under supervision. Potential complications can have serious and hazardous consequences for the patient during major bile duct injury. Substantial hands-on supervision during the initial phases of the learning curve, with gradual achievement of independence, is expected.

Laparoscopic procedures have a complex learning curve that affects clinical and financial outcomes [5,8]. However, expertise in laparoscopic surgery is not readily available, posing a challenge for resident training, particularly in smaller hospitals with limited surgical case volume. These factors call for optimizing the training period for surgical residents. There is some evidence supporting the cost-effectiveness and safety of ST systems [9].

Despite significant technological advances and growing experience with telecommunication and telementoring in health care, the evidence base for educational outcomes remains limited [10-12]. A systematic review by Erridge et al [13] found that, although telementoring demonstrates feasibility and safety, most studies lack rigorous comparative designs to evaluate skill acquisition. A subsequent systematic review from December 2023 by Fernandes et al [14] examined the educational outcomes of surgical telementoring (ST) during surgery. Using ST as the intervention in a continuous professional development program, the authors used the Moores Outcome Framework to evaluate educational outcomes [15]. They reported a lack of high-quality research, such as randomized controlled trials (RCTs), to elucidate the causal effect of ST on educational outcomes.

Furthermore, Chepkoech et al [16] reviewed telementoring systems for surgical training in low- and middle-income countries. They called for standardized qualitative and quantitative metrics, embedded within well-defined training frameworks, to assess the long-term effects of ST on surgical competence and to enable reproducibility and cross-study comparisons.

The Laparoscopic Colorectal (Lapco) Training the Trainers (TT) program, developed in the United Kingdom, provides a validated framework for standardized surgical mentoring and communication. The program established structured communication protocols between the mentor (trainer) and mentee (trainee) during Lapco surgery [17,18]. These principles have been adopted internationally, including in the Norwegian adoption (LapcoNor), to ensure consistent, high-quality intraoperative teaching.

Initiated in 2014, LapcoNor was a collaboration between the Norwegian Health Authority (South East) and Lapco International UK to standardize training in Lapco surgery in Norway [19]. The Norwegian program was described in detail in the Norwegian surgical journal, *Kirurgen* [20]. Its standardized communication framework provides an ideal foundation for ST, where a clear, structured dialogue is essential to overcome the physical separation between mentor and trainee. The Lapco TT program, adapted to Norway by the LapcoNor group, was used exclusively to prepare and train the involved mentors prior to participation. LapcoNor principles served as the intraoperative communication framework during resident supervision of surgical procedures.

Multimedia Appendix 1 describes how the LapcoNor group supervised the included telementors and on-site mentors to integrate the communication principles described by Mackenzie et al [17] into the study.

While Lapco principles were used at the micro level to facilitate concise verbal communication and enhance technical competency by addressing surgical challenges, the GROW (goals, reality, options, wrap-up) model was used for surgical coaching [21,22]. Surgical coaching has emerged as an effective method for developing surgical competence and will be implemented using the GROW model. Unlike mentoring, surgical coaching using the GROW model provides a macrostructure for verbal interactions during the telementoring session. It may facilitate the mentee’s ability to make independent decisions, thereby evolving both technical and nontechnical surgical skills. A description of the GROW model of surgical coaching, adapted for telementoring sessions, is provided in Multimedia Appendix 2.

There has been a focus on procedure-specific metrics to structure surgical education and assessment, minimizing subjectivity in the trainee-trainer relationship. van Zwieten et al [23] presented a systematic review of assessment methods in Lapco surgery. They differentiated between procedure-specific and general rating scales for the objective assessment of technical skills, enabling accurate feedback in a training environment.

**Figure 1.**
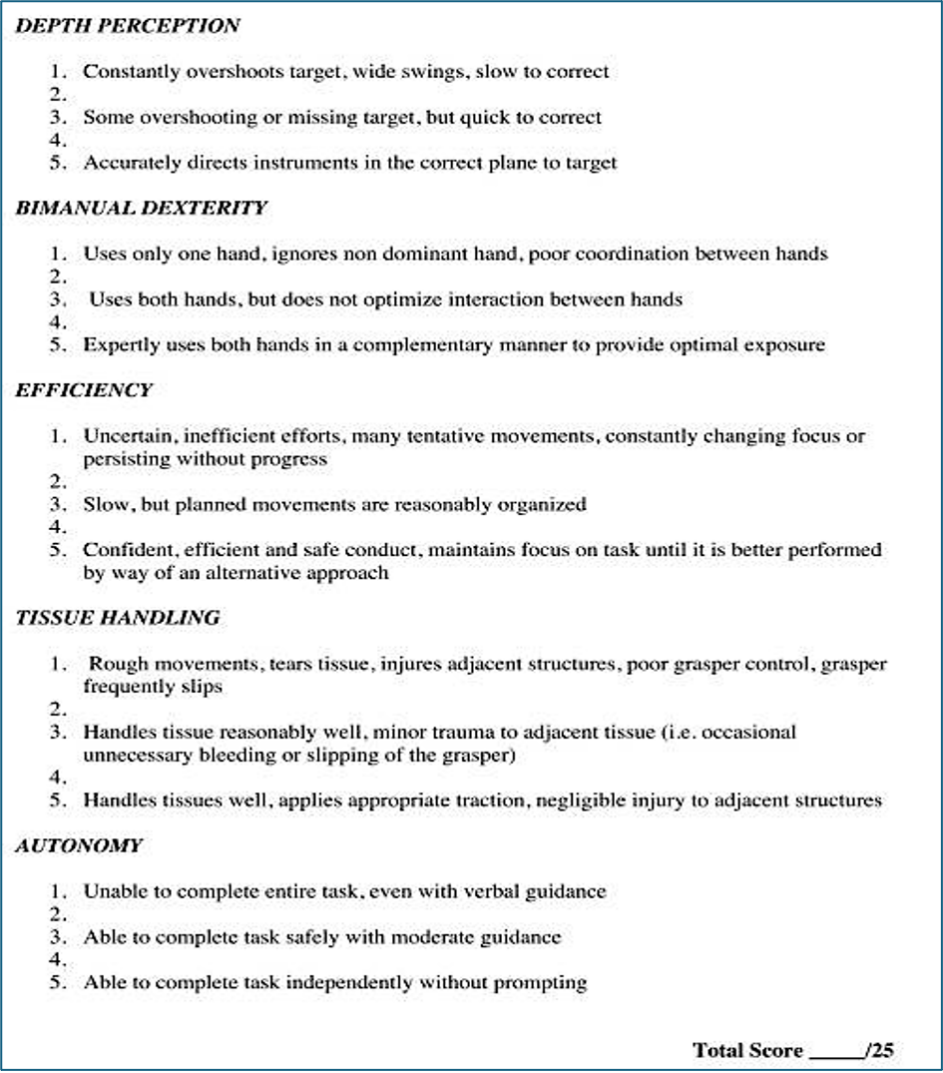
GOALS (Global Operative Assessment of Laparoscopic Skills). The assessed parameters and the scoring system are shown.

This study applies Lapco communication principles and standardized coaching using the GROW model in the intervention arm, thereby minimizing communicative bias while evaluating the educational efficacy of the telementoring modality. Using the Global Operative Assessment of Laparoscopic Skills (GOALS) score, the most widely used general rating system for laparoscopic procedures, the study enables comparison with existing and future literature. The GOALS score, including its parameters and scoring systems, is shown in Figure 1.

Given the increasing reliance on remote surgical education following the COVID-19 pandemic, there is a need for RCTs to assess ST as an intervention, enabling the acquisition of technical and nontechnical surgical skills to improve individual surgeons’ performance and patient outcomes. An RCT of this caliber will inherently pose strategic and practical challenges that require resources, which might be wasted due to poor study design [24]. Pilot trials are recommended as an essential initial step in assessing the feasibility of a full-scale RCT [25].

ST may offer several advantages over traditional hands-on mentoring: (1) the telementor shares the same visual perspective as the operating resident without competing for the operative field; (2) the structured communication required in telementoring may enhance deliberate feedback; (3) the combination of real-time guidance with systematic postoperative coaching using the GROW model may reinforce learning more effectively than traditional, unstructured mentoring; and (4) the telementor can provide undivided attention to teaching without the distraction of potentially needing to take over the procedure.

This pilot RCT aims to provide foundational data for a future full-scale trial. The study aims to evaluate whether real-time ST, combined with structured postoperative coaching, is superior to traditional on-site mentoring for developing surgical skills among surgical residents. Our primary hypothesis is that residents receiving telementoring will show a 3- to 5-point improvement in GOALS scores compared with the control group.

**Figure 2.**
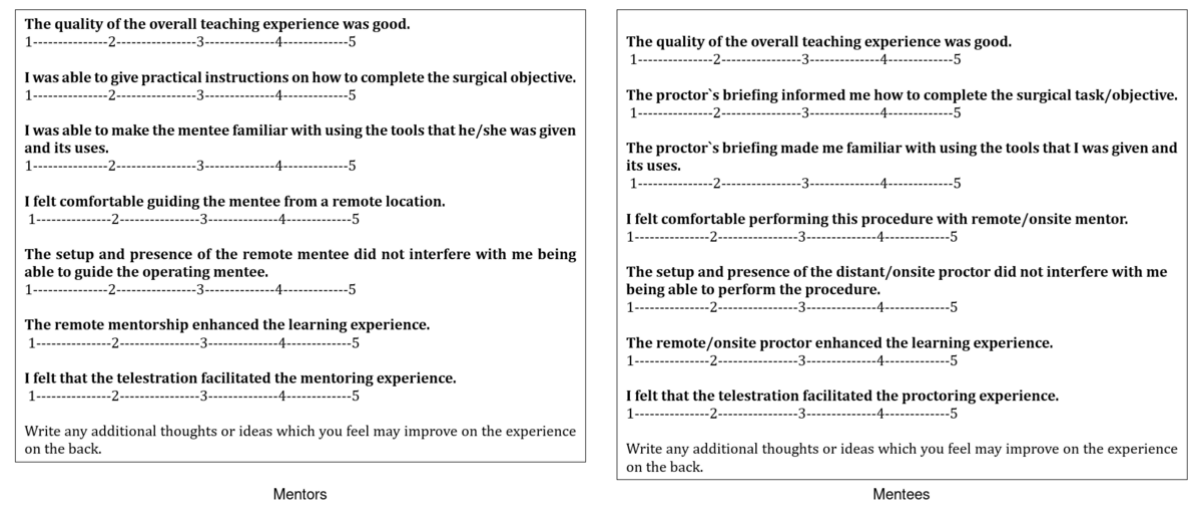
Resident and mentor satisfaction survey to obtain satisfaction scores from both the intervention and control arms of the study. The satisfaction survey used a 5-point Likert scale, with 1=strong disagreement and 5=strong agreement.

Secondary objectives include assessing Non-Technical Skills for Surgeons (NOTSS) score, participant satisfaction, and feasibility outcomes. Table 1 presents the NOTSS score, including their parameters and scoring system, and Figure 2 presents the scoring systems used to obtain satisfaction scores from the involved mentees and mentors in both the intervention and control arms.

**Table 1.** The NOTSS (Non-Technical Skills for Surgeons) score.

Category and elements	Rating^a^
Situation awareness	
Gathering information	1, 2, 3, 4
Understanding information	1, 2, 3, 4
Projecting and anticipating future state	1, 2, 3, 4
Decision-making	
Considering options	1, 2, 3, 4
Selecting and communicating options	1, 2, 3, 4
Implementing and reviewing decisions	1, 2, 3, 4
Communication and teamwork	
Exchanging information	1, 2, 3, 4
Establishing shared understanding	1, 2, 3, 4
Coordinating team activities	1, 2, 3, 4
Leadership	
Setting and maintaining standards	1, 2, 3, 4
Supporting others	1, 2, 3, 4
Coping with pressure	1, 2, 3, 4

a 1: poor, 2: marginal, 3: acceptable, 4: good.

This pilot study will specifically (1) evaluate the feasibility of recruitment, randomization, and intervention delivery; (2) assess the acceptability of the telementoring system among residents and mentors; (3) identify logistical challenges requiring modification before a definitive RCT; and (4) provide preliminary effect size estimates to refine sample size calculations for a future full-scale trial.

## Methods

### Study Design and Setting

This study is a parallel, 2-group, assessor-blind, multicenter pilot RCT. Participating surgical residents are allocated in a 1:1 ratio to either the intervention group, which receives real-time intraoperative telementoring and postoperative coaching, or the control group, which receives traditional intraoperative mentoring. The intention-to-treat principle will be applied. The study adheres to the latest SPIRIT (Standard Protocol Items: Recommendations for Interventional Trials) 2025 statements recommended for RCT protocols, as shown in Checklist 1 [26].

The Northern Norwegian Health Care Trust comprises 11 hospitals, including 1 university hospital and 10 local hospitals. Four hospitals within the Northern Norwegian Health Care Trust were initially assessed for eligibility as study sites: Sandnessjøen, Mo i Rana, Stokmarknes, and Bodø. Following a feasibility assessment, 2 hospitals—Sandnessjøen and Mo i Rana—were selected as active study sites based on surgical volume, resident availability, geographic location for ease of patient and resident inclusion in the study, and telementoring infrastructure, including the availability of telementors and on-site mentors. Stokmarknes and Bodø were not included as active recruitment sites due to anticipated pressure from acute cases in these locations, which would affect the elective surgical plan that the telementoring project was part of. Additionally, one of the initially included telementors based in Stokmarknes had to withdraw from the study. Although the telementoring infrastructure was initially installed at these locations to support patient and resident inclusion in the study, we ultimately decided to restrict inclusion facilities to Mo i Rana and Sandnessjøen. This facilitated optimal collaboration among actively participating telementors, residents, and the project administrator, KB, who was located in Bodø. Depending on the results of this pilot study, the expansion to include Stokmarknes and Bodø in a definitive RCT will be considered.

For this pilot RCT, 2 of the local hospitals, Sandnessjøen and Mo i Rana, served as study sites. Both locations offer surgical residency training programs that provide LC training.

### Technical Setup of the Telementoring System

Both hospitals, including patients in the study, have implemented ST systems in the ORs. The Olympus telecollaboration platform, MedPresence, provides a telementoring system that uses voice instructions delivered via headphones to assist the mentee throughout laparoscopic surgery. Simultaneously, the telementor observes the procedure in real time via a live stream from the laparoscopic camera. Telestration may be used, at the request of the telementor or the telementee, to provide annotations on a static picture of the ongoing surgical procedure. The telestration session begins when the telementor instructs the mentee to cease surgery and directs their attention to the visual aspect. Telementors may demonstrate surgical planes and strategize dissection.

The telementor may alternate between the external camera, which provides an overview of the laparoscopic trocars and the spatial positions of the OR and anesthesia nurse, and the laparoscopic camera. The system captures audio from the mentor-mentee dialog and footage from the laparoscopic surgery and the external camera. The external camera and its audio recording provide a foundation for assessing interactions between the telementee and OR staff, enabling the evaluation of the telementee’s nontechnical skills.

In both study arms, the standard operative team includes the operating resident, a camera-holding assistant (assisting surgeon), a scrub nurse, and a circulating nurse.

**Figure 3.**
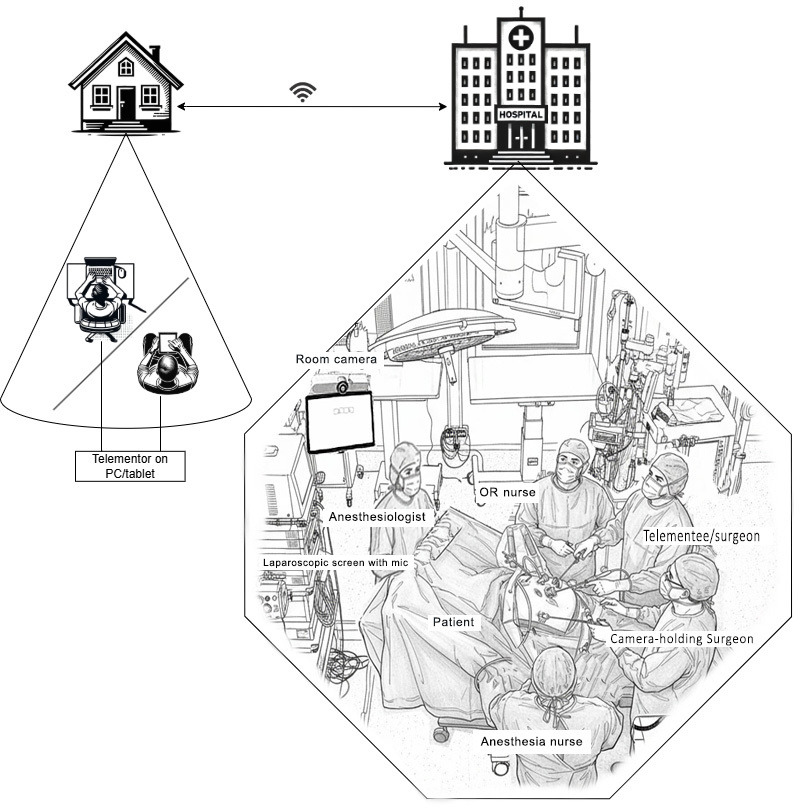
Configuration of the operating room (OR) setup during telementored sessions in the intervention group. Participating personnel include a resident, a camera-holding surgeon, a scrub nurse, a telementor (remotely located), an anesthesia nurse, and an anesthesiologist. Note the small microphone placed beside the soundbar, beneath the screens displaying the laparoscopic camera images.

In the intervention group, the local assisting surgeon, an experienced laparoscopic surgeon, holds the laparoscopic camera and assists the operating resident solely by moving the camera to meet the resident’s demands, providing instructional guidance only when patient safety requires it. The camera-holding surgeon’s role is limited to emergency assistance. In the control arm, the on-site mentor, an experienced laparoscopic surgeon, is scrubbed and provides active instruction throughout the procedure, while the same support personnel configuration is maintained in the OR. Figure 3 shows the intraoperative setup during a telementored procedure in the intervention arm.

In the control arm, the Lapco principles of intraoperative communication and the GROW model are not applied during the mentor’s preoperative, intraoperative, and postoperative guidance of the mentee.

### Eligibility Criteria: Residents, Mentors, and Patients

#### Residents

The eligibility criteria for the residents are as follows:

- General surgery residents in years 1 to 6 of their specialty education who have partially or completely performed more than 10 laparoscopic procedures
- Stratification according to experience
- Having completed the mandatory national course on general laparoscopic principles and the mandatory LC course
- Agreement with the mentor on the communication model during surgery. This model is derived from the LapcoNor principles [17]. Residents in the intervention group received an introduction to communication principles during telementoring and an orientation to the telementoring equipment used in the OR.

#### Mentors

The eligibility criteria for the mentors are as follows:

- Consultant surgeon with more than 5 years of experience performing LCs independently
- Acquainted with the assessment of videos for the GOALS score [27]
- Both telementors and on-site mentors had to attend the national LapcoNor TT course and follow standardized communication norms with the mentee during surgical mentoring, thus diminishing the bias of communicative difference.

#### Patients

The eligibility criteria for the patients are as follows:

- Gallstone disease without a clinical history of cholecystitis
- BMI <38
- No previous history of upper abdominal laparotomy
- No previous history of percutaneous gallbladder drainage
- Patient provided informed consent

Each telementoring procedure will be followed by a coaching session in which the mentee describes their performance (an opportunity for self-reflection). The telementor will provide performance-enhancing feedback to formulate a take-home message. Both the GROW model and the Lapco TT model will be used in telementoring sessions as macro- and micromodels for surgical guidance.

Residents will continue their standard surgical training during the study period. No restrictions will be imposed on other educational activities (eg, simulation training and courses). To promote participant retention, procedures will be scheduled within a 3-day block to minimize scheduling conflicts. Residents who cannot complete all 5 procedures will be followed for available outcomes. Reasons for withdrawal will be documented.

### Recruitment Procedures

The residents will be randomized into 2 groups. Residents in the control group will receive traditional OR supervision throughout all 5 consecutive procedures, with on-site mentoring by an experienced surgeon. The intervention group will receive guidance from a remotely located expert via live stream during 5 consecutive procedures.

Recruitment of research participants (surgical residents) will primarily occur at their local hospitals. During 2024 and 2025, all patients referred to hospital surgical departments with a diagnosis of gallstone disease and scheduled for LC will be assessed for inclusion. Study residents will be scheduled for 5 LCs within 3 days. If not enough residents are recruited at the local hospitals, recruitment efforts will be extended to include residents from other hospitals.

### Allocation and Blinding

The randomization process will use a predetermined statistical sequence generated by an external researcher who was not directly engaged in the study. The allocation sequence will be concealed using a centralized web-based randomization system. Personnel enrolling participants will not have access to the sequence until after enrollment. All individuals who actively participate in the experiment will be granted access to the allocation codes after randomization. Residents are enrolled by KB. After enrollment confirmation, the allocation is revealed by KB.

Computer-generated stratification will be used for randomization to create balanced and unpredictable groups. To ensure balance across groups with respect to significant confounders, the randomization process will be stratified by trainees’ experience, explicitly distinguishing between those who had performed 5 to 10 LCs and those who had performed 10 to 20.

### Participant Timeline

**Figure 4.**
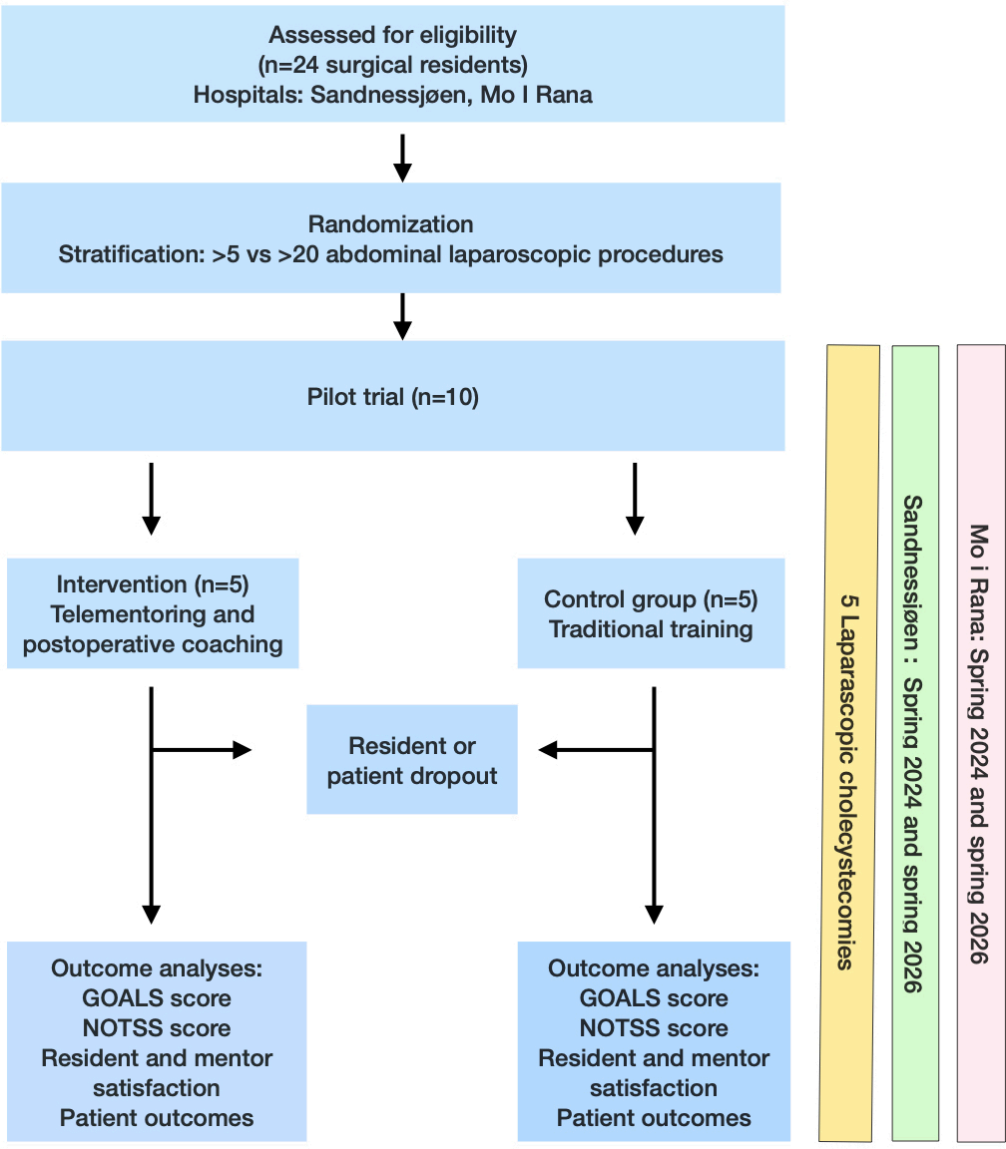
Design of the SURGTEACH (Surgical Telementoring in Acquisition of Surgical Skills of Laparoscopic Cholecystectomy) trial: CONSORT flow and participant timeline. The intention-to-treat (ITT) principle will be applied. CONSORT: Consolidated Standards of Reporting Trials; GOALS: Global Operative Assessment of Laparoscopic Skills; NOTSS: Non-Technical Skills for Surgeons.

To ensure transparency and adherence to reporting standards for RCTs, according to the Consolidated Standards of Reporting Trials (CONSORT), the trial design is summarized in the CONSORT flowchart in Figure 4. The timeline of surgical residents’ inclusion in both groups is shown. Residents who consented to inclusion will be informed of randomization 1 week before the planned procedures. In addition to the planned stepwise procedure description, standardized information on communication during the telementoring procedure will be provided.

This protocol follows the SPIRIT 2025 guidelines for clinical trial protocols. A complete SPIRIT checklist is provided as Checklist 1.

### Blinding

Blinding the trainees is not feasible given the characteristics of the intervention. Nevertheless, the evaluators who assessed performance needed to be unaware of the assignment of individuals to specific groups. Given the nature of the intervention, unblinding is not applicable to participants. For outcome assessors, any unintentional unblinding would be documented. No emergency unblinding procedures are required because the intervention poses no direct safety risk.

### Interventions

A skilled surgeon will serve as the camera-holding assistant for all procedures in the intervention group. They will intervene only at the time of unexpected events during surgery requiring emergency on-site assistance. Second, residents in the intervention group will receive postoperative coaching grounded in Lapco TT principles and the GROW model. In contrast, the control group will follow standard, unstructured guidance during the procedure. The mentors and mentees will sign a contract accepting the educational setting and the prerequisites before participating in the trial. Table 2 summarizes the intervention and control conditions.

**Table 2.** Comparison of intervention and control groups.

Component	Telementoring group (intervention)	Traditional training group (control)
Mentor location	Remote via secure video link. May use a laptop or other mobile devices	Physically present in operating room
Verbal communication mode	Headphones with a mic during surgery for real-time mentor-mentee communication	Direct verbal communication; physical gestures
Visual guidance	Live stream of procedure. Possibility for static image annotation (telestration); verbal description of dissection planes	Direct pointing; hands-on demonstration capability
Mentor’s view	Laparoscopic camera feed + external OR^a^ camera.	Direct laparoscopic monitor view + peripheral vision of OR
Physical intervention	Not possible (local consultant available for emergencies)	Can take instruments to demonstrate; tactile guidance
Postoperative coaching	Mandatory structured GROW^b^ model session (15‐20 min)	Standard informal feedback (unstructured, variable).
Documentation	All sessions recorded; coaching documented	Standard operative notes only.

a OR: operating room.

b GROW: goals, reality, options, wrap-up.

### Intervention Group (Telementoring)

The telementoring protocol comprises 3 main phases: preprocedural setup and coaching, intraoperative telementoring, and postoperative coaching.

#### Preprocedure Setup and Coaching

Before commencing the surgical procedure, a standardized setup phase of approximately 10 to 15 minutes is implemented. Key steps to ensure operational readiness include the following: the resident confirms connectivity to the telementoring system with the remote mentor; a bilateral communication check of the audio headset is performed, and the video feeds from both the laparoscopic and external OR cameras are verified by the telementor. This phase concludes with a brief preoperative discussion to review the surgical plan and anticipate potential challenges.

#### Intraoperative Telementoring

During the operation, the telementor provides real-time guidance by observing a high-definition video stream and maintaining continuous audio communication via a wireless headset. When anatomical clarification is required, the telementor activates the telestration mode. This process involves the following:

- The procedure is temporarily paused using a specific “stop word” command from the mentor.
- A static image is captured from the laparoscopic feed.
- The mentor annotates the image using lines, circles, or arrows to highlight relevant anatomical structures, dissection planes, or areas of concern.
- The resident confirms their understanding of the annotations before the procedure is resumed.

The Lapco communication protocol, an integral component of the Lapco standardized teaching framework, follows the “6 steps”: stop → identify → explain → instruct → check → assess. The steps are defined as follows:

1. Stop. The telementor (trainer) initiates the sequence by pausing the procedure completely with the stop signal, often when the telementee (trainee) encounters difficulty or reaches a critical juncture, to gain the telementee’s full attention and prevent errors.
2. Identify. The telementor asks the telementee to clearly identify the specific problem, concern, or relevant anatomical structure to ensure mutual understanding of the issue.
3. Explain. The telementor provides a brief explanation of the underlying principle or rationale behind the next required action, connecting theory to practice.
4. Instruct. Clear, concise instructions are provided to the telementee on how to perform the next step correctly.
5. Check. The telementee verbally confirms that they understand the instructions and are prepared to execute them safely by repeating the instructions provided in step 4.
6. Assess: After the telementee attempts the maneuver, the telementor provides an assessment and determines whether the procedure can proceed safely without further instructions or whether the on-site surgeon should assume control of the procedure. The latter should be avoided to maximize learning.

A local consultant surgeon is scrubbed for all procedures and assists as the camera-holding surgeon during the laparoscopic procedure but does not provide teaching input unless patient safety requires intervention.

#### Postoperative Coaching (10-15 Minutes Within 1 Hour of Procedure Completion):

A structured coaching session is conducted postoperatively, within 1 hour of procedure completion, and lasts approximately 10 to 15 minutes. This session uses the GROW model and is conducted via a videoconferencing platform.

The process begins with the resident completing a self-assessment of their performance, focusing on their initial goals and the actual surgical outcomes (goals and reality). Subsequently, the mentor provides constructive feedback, referencing specific procedural moments observed during the operation. The session concludes with the joint identification of specific action points for improvement (options and wrap-up). Finally, the key learning points generated during the session are formally documented for future reference and to support the continuity of training.

### Control Group (Traditional Training)

The control group operates within the framework of conventional surgical training protocols, characterized by direct, in-person mentorship.

#### Intraoperative Mentoring

During the surgical procedure, an experienced consultant surgeon is physically scrubbed in alongside the resident. The primary mode of instruction is direct verbal guidance throughout the operation. Mentors retain discretion to physically interact during the case. They may guide the resident’s hands, indicate anatomical structures on the surgical screen, or temporarily assume control of instruments to demonstrate a specific technique. While the same standardized procedural steps are typically followed, the mentor provides teaching and feedback at their discretion, in the absence of a formalized, structured teaching framework.

#### Postoperative Feedback

Postoperative feedback in this group consists of a standard informal debriefing, conducted solely at the mentor’s discretion. This approach is defined by the absence of mandated structure, specific timing requirements, or formal documentation requirements. This methodology represents the current standard practice in many surgical training environments.

### The Surgical Procedure

The LC procedure was standardized using a stepwise approach, incorporating the principles of creating the critical view of safety before transecting any structure in Calot triangle [28,29]. All included residents were instructed in intraoperative communication in accordance with Lapco and GROW principles. A step-by-step procedure was provided for both mentees and mentors, and adherence was required. A detailed description of the step-by-step surgical procedure is provided in Multimedia Appendix 3.

### Outcomes

#### Primary Outcomes

The primary outcomes are the GOALS and NOTSS scores (Figures 1 and 2). The GOALS score is the only global rating scale developed and validated for assessing laparoscopic surgery. Five subscales are assessed: depth perception, bimanual dexterity, efficiency, tissue handling, and autonomy. Each subscale is scored from 1 to 5, yielding a maximum score of 25 when assessing the resident’s laparoscopic technical skills. The NOTSS score assesses the operating surgeon’s nontechnical surgical skills by evaluating defined parameters [30].

The NOTSS score will be derived from video recordings of interactions between the operating telementee and the rest of the OR team. A 3-member evaluation group of specialized OR nurses will be established to assess the NOTSS score for each procedure by reviewing video footage from the room camera.

The GOALS score will be assessed by video recording the laparoscopic camera footage of each procedure performed by the included registrars. A 3-member evaluation group of expert laparoscopic surgeons will be established to assess the GOALS score for each procedure by reviewing the laparoscopic video footage, including the verbal communication between the mentee and the mentor.

Both evaluation groups will be presented with video footage of each included mentee in a random order to obtain blinded evaluations of the GOALS and NOTSS scores.

#### Secondary Outcome Measurements

##### Satisfaction Scores

Residents in both groups completed a satisfaction questionnaire after each procedure, allowing them to self-assess their satisfaction with the learning outcomes.

The satisfaction survey used a 5-point Likert scale, with 1 indicating strong disagreement and 5 indicating strong agreement. A similar satisfaction survey was administered to the mentors in each group.

##### Patient-Related Outcomes and Complications

Intraoperative unfavorable incidents will be classified according to the Oslo Classification of Intraoperative Unfavorable Incidents [31].

- Grade 1: minor incidents with no consequences (eg, minor bleeding controlled immediately and small gallbladder perforation with minimal spillage)
- Grade 2: incidents requiring intervention but not affecting the procedure’s outcome (eg, moderate bleeding requiring additional hemostatic measures and bile spillage requiring irrigation)
- Grade 3: incidents significantly affecting the procedure (eg, conversion to open surgery, common bile duct injury, and bowel injury).

This classification complements the Clavien-Dindo Classification, which captures postoperative complications [32]. By applying both systems, a comprehensive assessment of intraoperative and postoperative morbidity will be achieved. Interventions by the attending consultant surgeon and conversions to laparotomy will also be assessed.

### Sample Size

This pilot RCT is designed to identify challenges and unforeseen pitfalls in conducting a proper RCT. Given the novelty of the topic and the resources required for an RCT, it is necessary to conduct a pilot study to assess the feasibility of a full-scale RCT.

The full RCT is designed as a superiority trial to demonstrate the superiority of telementored resident skill acquisition in the OR compared with standard training methods. Previous investigations have shown that the SD of the GOALS score is 2.5. We hypothesize that the intervention group will demonstrate significant clinical improvement in skills, as evidenced by a 3‐ to 5-point increase in the GOALS score relative to the control group. To achieve a statistical power of 0.8, a significance level (*P* value) of <.05, and to account for an expected dropout rate of 20%, 12 residents per group are required for an RCT.

This pilot study will include 5 residents in both the experimental and control arms (n=10). Residents will be stratified into 2 groups according to the number of prior LCs.

### Data Collection and Management

Each video recording will be stored in the OR recording system. The video recordings will be edited by KB (corresponding author) into predefined segments of the surgical procedure, as specified in the stepwise procedure description. The edited video recordings will be annotated. These standardized, edited recordings will be anonymized and randomized for each resident and distributed to the evaluation group for assessment of GOALS and NOTSS scores.

The project secretary will provide each evaluation group with a Questback questionnaire to collect their assessments. The project secretary, who holds the anonymization and randomization key, will then collect and decode the assessments.

Data will be collected and managed using REDCap, hosted at the University Hospital of Northern Norway [33]. Data items (resident satisfaction and mentor satisfaction) will be collected immediately after each procedure. Given the pilot nature of this study, no formal steering committee or data safety monitoring board was established.

**Figure 5.**
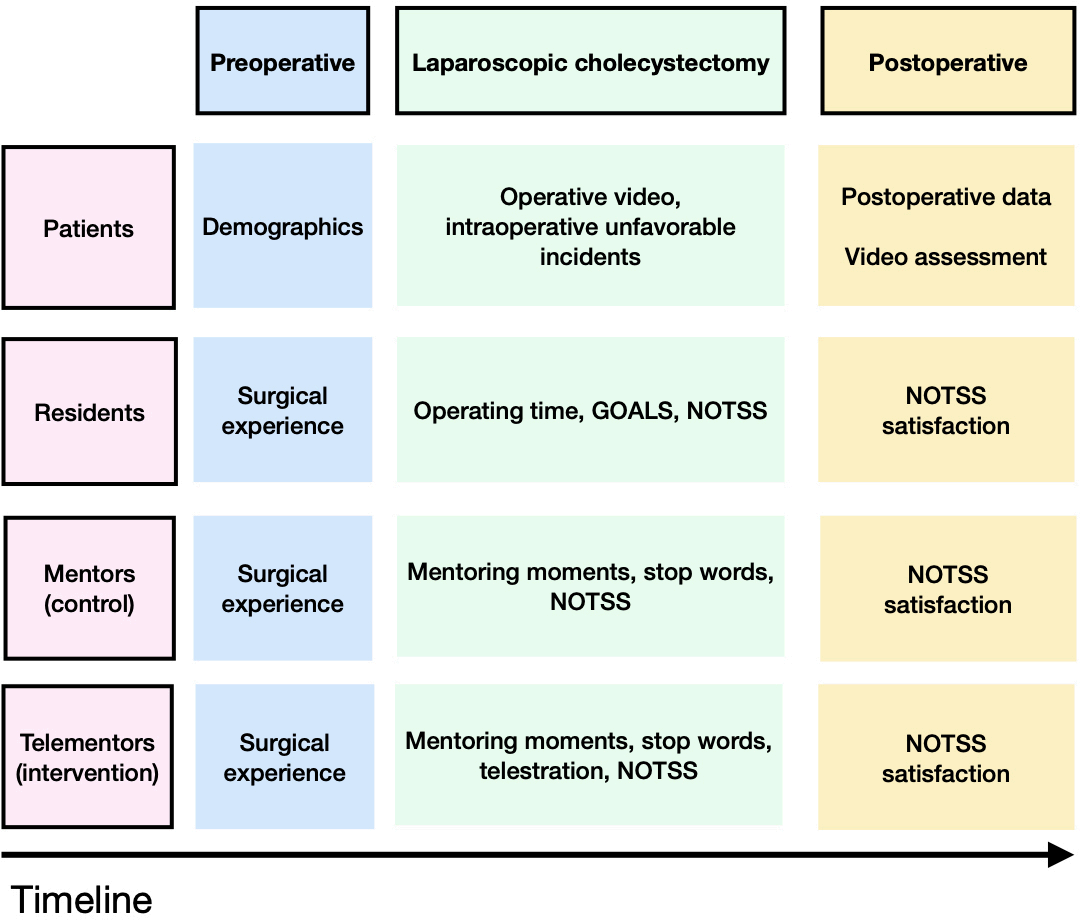
Trial data collection timeline. GOALS: Global Operative Assessment of Laparoscopic Skills; NOTSS: Non-Technical Skills for Surgeons.

GOALS and NOTSS scores will be collected by giving the mentor group 1 month to assess the video records. Patient-related data will be collected retrospectively by reviewing the electronic health record. The timeline of data collection is shown in Figure 5.

### Statistical Analysis

The primary analysis will compare mean GOALS scores between the intervention and control groups. An independent-samples *t* test will be used for this comparison, provided that the assumption of normality is met; if normality is violated, the nonparametric Mann-Whitney *U* test will be applied. Effect sizes will be calculated using Cohen *d* to quantify the magnitude of the difference between groups and to inform sample size estimation for a future definitive RCT.

Secondary analyses will include comparisons of NOTSS scores between the intervention and control groups using the same statistical approach as that used for the primary outcome. Within-group improvement in technical performance across the training period will be assessed by comparing GOALS scores from the first to the fifth procedure using paired *t* tests. Participant satisfaction scores will be compared between groups using the Mann-Whitney *U* test, as these data are collected on an ordinal Likert scale.

Multivariate analyses will be conducted using linear regression models to examine the association between group allocation and GOALS scores while adjusting for potential confounding factors. Group allocation (intervention vs control) will be included as the primary predictor. Covariates will be selected a priori based on theoretical relevance and will include baseline surgical experience (defined as completion of 5‐10 vs 10‐20 prior LCs), baseline GOALS score from the first procedure, year of residency training, study site (Sandnessjøen vs Mo i Rana), and indicators of procedure complexity, including intraoperative findings and operative time.

Exploratory analyses will be performed to further characterize performance development and relationships between outcomes. Learning curves across the 5 procedures will be evaluated by examining trajectories of GOALS scores using repeated-measures ANOVA or mixed effects models, depending on the data structure and model assumptions. Subgroup analyses stratified by baseline experience level will be undertaken, and correlations between GOALS and NOTSS scores will be assessed to explore the relationship between technical and nontechnical performance.

As this study is a pilot trial, feasibility outcomes will be summarized descriptively. These outcomes will include the recruitment rate, expressed as the number of participants enrolled per month; balance between randomized groups; adherence to the study protocol (including completion of all 5 procedures and scheduled coaching sessions); the frequency and nature of technical failures related to the telementoring system; and withdrawal rates, along with reasons for withdrawal. Participants may withdraw from the study at any time. In cases where a resident discontinues the intervention, outcome data will be collected, where feasible, and analyzed using the intention-to-treat principle.

### Data Presentation

Continuous variables will be presented as mean (SD) for normally distributed data and as median (IQR) for skewed distributions. Categorical variables will be presented as frequencies and percentages. A *P* value <.05 will be considered statistically significant. Given the pilot nature, emphasis will be placed on effect sizes and CIs rather than *P* values alone. Descriptive statistics and data analysis will be performed using Microsoft Excel - Office 365 and IBM SPSS Statistics software (version 31.0; IBM Corp).

### Data Monitoring

Data and study monitoring will be performed once 10 residents have been recruited to the pilot trial. Given the pilot nature and low-risk intervention, a formal data monitoring committee has not been established. The principal investigator will review safety data after every 5 residents. The principal investigator, the telementors, and the on-site consultant surgeon will conduct monitoring in brief meetings (telephone calls or Teams meetings) prior to the inclusion of each resident. Technical issues related to telementoring, the mentoring framework, and coaching will be addressed. Given the study’s novelty, adjustments to the telementoring framework may be necessary.

Given the pilot study’s intensive follow-up (5 procedures in 3 d), minimal missing data are expected. For the primary analysis, available-case analysis will be used. Descriptive patterns of missing data will be reported to inform the definitive RCT.

Trial monitoring will be conducted by the principal investigator. This includes a quarterly review of recruitment rates, protocol deviations, technical system performance, and adverse events. Given the single-site coordination and pilot scale, on-site monitoring visits are not planned.

### Ethical Considerations

The study was approved by the ethical committee (REK HELSE NORD 32592) and the data protection officer at Nordlandssykehuset Hospital Trust, Bodø, Norway.

All collected data were anonymized prior to analysis to ensure participant (patients and residents) confidentiality. Video recordings were deidentified and securely stored on password-protected institutional servers with access restricted to authorized personnel. Informed consent was obtained from all participants, including consent for recording and data use. The study complies with applicable data protection regulations and was approved by the relevant ethics committee and data protection authority as mentioned above.

Surgical residents will be informed that their participation is entirely optional, that there will be no repercussions if they choose to withdraw, and that they may withdraw without providing a reason. Upon signing in, all surgical residents must sign an electronic permission document and a mentee-mentor contract (intervention arm). Protocol adherence will be monitored by reviewing recorded sessions.

This educational intervention does not pose direct risks to participants beyond those inherent in standard surgical training. No specific posttrial care provisions are required. Residents receive no compensation for participation.

Patients will be asked to provide informed consent. The study group does not anticipate any ethical implications for patient participation, as the telementoring (intervention arm) will provide increased surgical expertise in the OR.

Any protocol modifications will be submitted to REK for approval before implementation. Amendments will be documented with date and rationale, communicated to investigators and the trial registry, and reported in the final publication.

### Patient and Public Involvement

This pilot study was designed without formal patient or public involvement in the research design, conduct, or reporting plans. Patients are included solely as surgical cases, with the study focusing on surgical trainee education rather than on patient-centered outcomes. Surgical residents, the primary study participants, were consulted informally during protocol development to assess the acceptability and feasibility of the telementoring intervention. For the planned definitive RCT, we will consider establishing a stakeholder advisory group, including patient representatives and surgical trainees, to inform outcome selection, dissemination strategies, and study conduct.

### Dissemination Plan

The results of this pilot study will be submitted for publication in an international peer-reviewed journal, regardless of the outcome (positive, negative, or inconclusive). Findings will be presented at relevant surgical education and telemedicine conferences. National and international conferences in 2026, focusing on the intersection of surgical education and telemedicine, which we are looking forward to participating in, include the following: (1) The Norwegian Surgeons' Annual Meeting, October 2026. Oslo, Norway; (2) AMEE Conference, August 2026, Vienna, Austria; and (3) ASiT Annual Conference, 2027.

The trial protocol and results will remain publicly accessible via ClinicalTrials.gov. If successful, we will engage with Norwegian surgical societies and health care policymakers to discuss implementation implications for rural surgical training programs.

## Results

### Preparations Before Onset of the Study

Before the trial began, all telementors and on-site consulting surgeons participated in a Lapco TT course. The configuration of the telementoring system was finalized in December 2023. All residents, consulting surgeons, and telementors were instructed on the setup and use of the telementoring system.

### Current Study Status and Timeline

#### Completed Activities

The project initiated its operations by configuring the telementoring system across all study sites by December 2023. Following this, all mentors received Lapco TT training in February 2024. Ethics approval was subsequently secured from the Regional Committee for Medical and Health Research Ethics (REK nord 32592) on October 23, 2023, and the most recent update of the trial was registered in ClinicalTrials.gov (NCT06421584) on December 8, 2024.

#### Recruitment Status

Recruitment commenced in the Spring of 2024. Initially, the MedPresence platform was used for the first 5 resident inclusions. Due to changes in the Olympus portfolio of telementoring systems and global partnership agreement with Proximie, a platform transition to Proximie occurred in July/August 202. This included training telementors on the new system in August 2025. By December 2025, 3 residents had been included using the new platform. The remaining recruitment is scheduled to start in February 2026.

#### Anticipated Timeline of the Pilot

The project anticipates completing all recruitment by May 2026, with data collection concluding in September 2026. The data analysis phase is slated for November 2026, and the manuscript submission for the final results is expected in January 2027.

## Discussion

### Principal Findings and Interpretation

We acknowledge that telementoring is often positioned as an alternative when on-site expertise is unavailable, and the intuitive assumption is that physical presence should be superior. However, our superiority hypothesis is based on several theoretical considerations.

First, telementoring may enhance the quality of verbal instruction. When the mentor cannot physically intervene, communication must be more precise and explicit, potentially improving the teaching dialog. The structured communication required by telementoring (stop-identify-explain-instruct-check-judge) enforces a pedagogical framework that may be applied less rigorously when mentors can simply take over the instruments.

Second, the mandatory structured postoperative coaching in the telementoring arm (using the GROW model) represents an educational enhancement not typically provided in traditional training. This combination of real-time guidance and systematic reflection may amplify learning beyond that achieved through informal feedback.

Third, the telementor maintains the identical visual perspective as the operating resident without competing for the operative field or camera control, potentially allowing for more focused observational teaching.

We acknowledge, however, that these are theoretical advantages that may not materialize in practice. The pilot study will test whether any educational benefit exists and, equally importantly, will provide data to refine or reject this hypothesis before investing in a larger definitive trial. If the pilot demonstrates no advantage—or disadvantage—for telementoring compared to on-site mentoring, this finding would itself be valuable, suggesting that telementoring can serve as an acceptable equivalent (noninferior) alternative when on-site expertise is unavailable.

### Anticipated Outcomes

This pilot RCT is designed to determine whether ST combined with structured postoperative coaching can enhance laparoscopic skill acquisition compared with traditional on-site mentoring. We anticipate that the structured nature of telementoring—requiring explicit verbal communication and systematic feedback—may enhance learning through increased deliberate practice and reflection. If our hypothesis is confirmed, we expect to observe a 3- to 5-point improvement in GOALS scores in the telementoring group, which would represent a clinically meaningful enhancement in surgical proficiency.

Equally important, this pilot will identify practical challenges in implementing telementoring for surgical education, including technological barriers, workflow integration issues, and participant acceptance factors that must be addressed before a definitive trial.

### Comparison to Prior Work

Previous telementoring studies have primarily focused on demonstrating technical feasibility and safety rather than educational outcomes [13,14]. The SURGTEACH (Surgical Telementoring in Acquisition of Surgical Skills of Laparoscopic Cholecystectomy) trial extends this work using validated educational assessment tools (GOALS and NOTSS) and a randomized design. The author group of this pilot RCT has previously published a prospective clinical trial evaluating the educational role of ST in minimally invasive surgery [34]. Unlike observational studies that compare outcomes before and after telementoring implementation, this parallel-group design with a concurrent control arm will provide more rigorous evidence of any educational benefit.

The integration of the LapCo TT framework distinguishes this study from prior telementoring research, which often lacked standardized communication protocols. By training all mentors—both telementors and on-site mentors—in identical communication principles, we minimize the confounding effects of differing teaching styles and isolate the effects of the mentoring modality itself.

### Strengths and Limitations

#### Strengths

This study represents the first randomized comparison of telementoring versus traditional training with a primary focus on educational outcomes. It employs validated, objective assessment tools, including GOALS and NOTSS, to ensure robust measurement of both technical and nontechnical skills. Outcome assessment is blinded through standardized video review, reducing the risk of assessor bias. Both study arms use a standardized communication framework, which minimizes variability in mentor–trainee interaction. In addition, postoperative coaching is delivered in a structured manner using the evidence-based GROW model. Randomization is stratified by trainee experience level, further strengthening internal validity and group comparability.

#### Limitations

As a pilot study, the relatively small sample size limits statistical power and precludes definitive conclusions regarding effectiveness. Blinding of participants is not feasible given the nature of the intervention, which introduces a potential risk of performance bias. The single-country setting may restrict the generalizability of the findings to other health care systems and training environments. The superiority hypothesis is based on an assumed benefit of structured telementoring that may not be fully realized in the final outcome assessment. Furthermore, a change in the technological platform during recruitment, from MedPresence to Proximie, may introduce heterogeneity in the intervention. The control group receives standard care, which may vary between mentors despite Lapco TT training, potentially increasing intermentor variability. Finally, assessing short-term outcomes limited to the first 5 procedures may not adequately capture long-term skill acquisition or retention.

### Future Directions

The results of this pilot study will directly inform the design of a definitive multicenter RCT. Specific aspects that may require modification include recruitment strategies, randomization procedures, outcome timing, and telementoring protocols. If substantial technical or logistical barriers are identified, alternative technological solutions will be explored before the definitive trial.

Beyond the immediate next steps, successful demonstration of telementoring efficacy could enable expansion to other laparoscopic procedures, support the development of national telementoring networks for rural surgical training, and inform educational policy for surgical residency programs in geographically challenging regions.

### Conclusion

The SURGTEACH trial is the first known randomized pilot trial to evaluate ST as an educational method. Globally and domestically, there are constraints on the number of surgeons available and strain on the surgical education system. The Norwegian health care system requires support to train sufficient surgeons, primarily due to geographical constraints and limitations in the education system. Therefore, there is a need for a fundamental change in surgical education, and ST has the potential to overcome these obstacles. This study has the potential to provide substantial evidence to enhance surgical education, particularly in rural health care facilities.

## Supplementary material

10.2196/73159 Multimedia Appendix 1 (a) Six-step Lapco-based trainer intervention of intraoperative teaching when a mentee is failing to progress. Adopted for telementoring sessions in the intervention arm of the study. (b) A structured, standardized framework for perioperative setup, dialogue, and closure of telementoring sessions, including performance-enhancing instructions and an opportunity for self-reflection for the telementee. The session is complete after the telementee formulates a take-home message in agreement with the telementor.

10.2196/73159 Multimedia Appendix 2 The adoption of the GROW model for telementoring procedures in the intervention arm of the study.

10.2196/73159 Multimedia Appendix 3 The standardized, detailed stepwise technique applied for laparoscopic cholecystectomy in both the intervention and control arms of the study.

10.2196/73159 Checklist 1 SPIRIT checklist 2025 for the SURGTEACH protocol. Note that the checklist is based on a replica of the manuscript presented in the supplementary documents, including line numbers in the document margins.

## Abbreviations

CONSORT: Consolidated Standards of Reporting Trials
GOALS: Global Operative Assessment of Laparoscopic Skills
GROW: goals, reality, options, wrap-up
Lapco: laparoscopic colorectal
LapcoNor: laparoscopic colorectal in Norwegian adoption
LC: laparoscopic cholecystectomy
NOTSS: Non-Technical Skills for Surgeons
RCT: randomized controlled trial
SPIRIT: Standard Protocol Items: Recommendations for Interventional Trials
ST: surgical telementoring
SURGTEACH: Surgical Telementoring in Acquisition of Surgical Skills of Laparoscopic Cholecystectomy
TT: Training the Trainers
