# Supplementary material for: Evaluating the Role of Surgical Telementoring in the Acquisition of Surgical Skills in Laparoscopic Cholecystectomy: Protocol for a Pilot Randomized Controlled Trial
Source: JMIR Res Protoc. 2026 Apr 17;15:e73159. doi: 10.2196/73159 (PMC13089673; doi:10.2196/73159)
Supplement: Multimedia Appendix 1 [file resprot-v15-e73159-s001.pdf]

Appendix 1:

a)

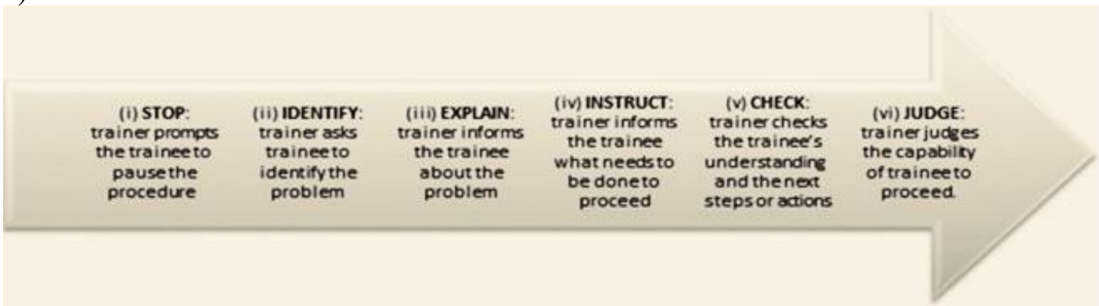

b)

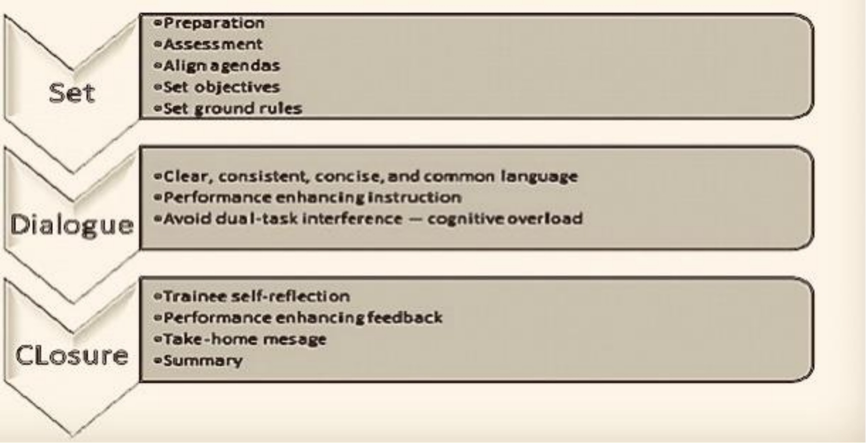

a) *Six-step Lapco-based trainer intervention of intraoperative teaching when a mentee is failing to progress.*
b) *A structured, standardized framework for perioperative setup, dialogue, and closure of telementoring sessions, including performance-enhancing instructions and an opportunity for self-reflection for the telementee. The session is complete after the telementee formulates a take-home message in agreement with the telementor.*
