# Supplementary material for: Evaluating the Role of Surgical Telementoring in the Acquisition of Surgical Skills in Laparoscopic Cholecystectomy: Protocol for a Pilot Randomized Controlled Trial
Source: JMIR Res Protoc. 2026 Apr 17;15:e73159. doi: 10.2196/73159 (PMC13089673; doi:10.2196/73159)
Supplement: Multimedia Appendix 2 [file resprot-v15-e73159-s002.pdf]

Coaching: The GROW model

The GROW model was used as a perioperative coaching framework to train surgical residents in the study's intervention group. This goal-oriented coaching model guides a coaching conversation through 4 stages:

1. Goals: focus on specific targets the resident wishes to achieve, defined in agreement with the mentor. Primarily formulated by the resident based on his/her understanding of the personal learning curve for the specific surgical procedure. Typically addressed prior to each surgical procedure in agreement with the telementor.
2. Reality: the mentor explores the true nature of the problem (a specific task during surgery or the target issue formulated in the 1st step of the GROW model) by analyzing the mentee's surgical performance during the live video feed (performance review).
3. Options: formulation of effective solutions, particularly to the issues that prevent the resident from achieving their goals. This is conducted through interactive dialogue and telestration (real-time on-screen drawing). Allows the trainee to explore different surgical approaches to overcome obstacles
4. Wrap-up: Conclusive debrief, including an examination of potential obstacles and strategies for overcoming them. Development of an action plan for candidates to move toward their originally stated goals.
