# Supplementary material for: Evaluating the Role of Surgical Telementoring in the Acquisition of Surgical Skills in Laparoscopic Cholecystectomy: Protocol for a Pilot Randomized Controlled Trial
Source: JMIR Res Protoc. 2026 Apr 17;15:e73159. doi: 10.2196/73159 (PMC13089673; doi:10.2196/73159)
Supplement: Multimedia Appendix 3 [file resprot-v15-e73159-s003.pdf]

Stepwise standardized technique of laparoscopic cholecystectomy

Patient position and port placement

a) The patient in a supine position, and the table is tilted anti-Trendelenburg and to the left to increase intraabdominal space in the right hypochondrium.

b) Two 12 mm ports and 2 five mm ports. A 30-degree camera is used.

- The camera port, 12 mm, is introduced in the umbilical area, preferably by a midline incision through the middle of the umbilicus, as the incision may be enlarged sufficiently to remove even a voluminous gallbladder without enlarging the incision outside the umbilical cavity.
- The second 12 mm port is introduced in the midline in the epigastrium, 3-5 mm below the xiphoid process. It should enter the abdominal cavity exactly in the angle between the falciform ligament and the anterior abdominal wall, with a direction towards the hilar region to avoid any direction vector in the port when working through the port.
- The two 5 mm ports should be parallel and directed somewhat cranially. The most medial of these two ports should be introduced 5 cm caudal to the gall bladder fundus. The lateral port is introduced 5-6 cm lateral to the former, with caution not to affect the right colon flexure immediately below (Figure 1)

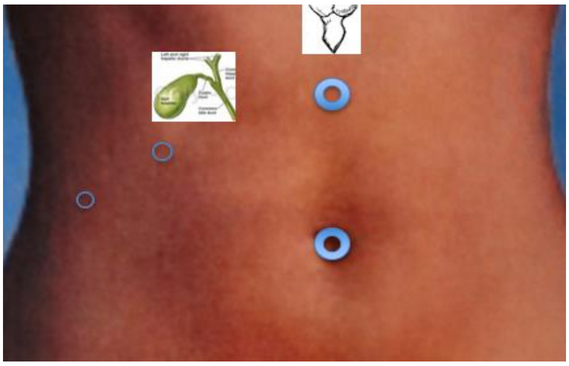

Figure 1. Port placement

Procedure principles

- **The main principles of safe dissection in laparoscopic cholecystectomy to diminish the possibility of the most serious complications of the procedure being bile duct injury:**

  A) Hilar anatomy orientation before any dissection is started

  B) Mobilization of the central 1/3 of the gall bladder to increase lateral infundibular traction, thus increasing the angle between the hepatic duct and the cystic duct (*critical view of safety*)

  C) All dissection of the cystic duct and the cystic artery starts at the infundibulum of the gall bladder and is then continued centrally to expose these structures.

Stepwise procedure:

1. **Lysis of adherences and exposure of the gallbladder**

a) The gall bladder fundus is retracted by a grasper in the lateral 5 mm port.

b) Any adhesions to the gallbladder are dissected close to the gall bladder wall to omit omental vessel bleeding, using scissors or a monopolar hook (Figure 2).

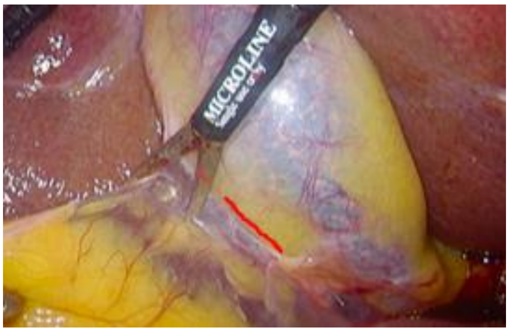

Figure 2.

Corpus dissection: monopolar diathermy/scissors

c) Any adhesions between the gall bladder infundibulum and the duodenum are divided without diathermy to avoid risk of stray current lesion of the duodenal wall (Figure 3)

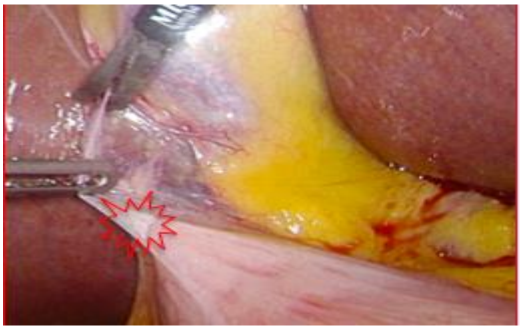

Figure 3.
Infundibulum: Cold scissors only

Hilar anatomy orientation before any dissection is started

d) The gall bladder infundibulum is retracted laterally, the left liver lobe is gently retracted. Then the hepatic artery, the common bile duct, the hepatic duct and the cystic duct are located. If not directly visualized, the surgeon should decide where these structures are located, before proceeding the operation (Figure 4).

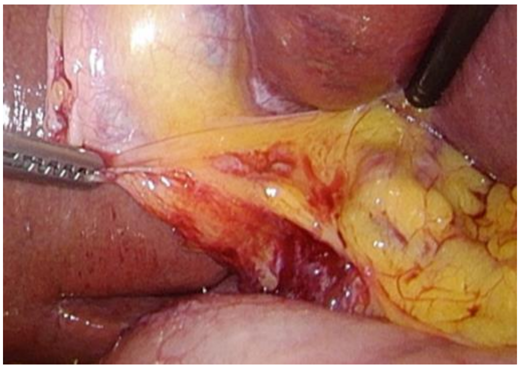

Figure 4.

Hilar anatomy orientation

Mobilization of the central 1/3 of the gall bladder

e) The dissection starts at the infundibulum, 7-8 mm distal to the most central margin of the infundibulum which can be seen in fig 3. This maneuver is required to be sure not to affect any tubular structure.

f) Dissection is continued along the medial border of the gall bladder for 5-6 cm, about 1 cm from the liver tissue, only the serosa is divided not to affect the cystic artery.

g) A similar incision is made in the serosa along the lateral side of the gallbladder. Sometimes this may be difficult due to a stone in the infundibulum, but it is always possible without dislocating the stone towards the gall bladder lumen, which usually is difficult.

h) A window is dissected behind the distal 1/3 of the gallbladder by sharp dissection with the hook or blunt dissection with the irrigation/suction instrument, a dissecting instrument may also be useful.

i) The dissected window may be enlarged by using two instruments in the opening, one pressing towards the fundus and the other towards the infundibulum, thus causing no traction onto the cystic duct.

j) The infundibulum is now retracted laterally. The described mobilization of the central part of the gall bladder, makes this retraction much more efficient, thus increasing the angle between the hepatic duct and the cystic duct substantially, making the subsequent dissection of the cystic duct and artery safer as the distance to the bile ducts is increased (Figure 5)

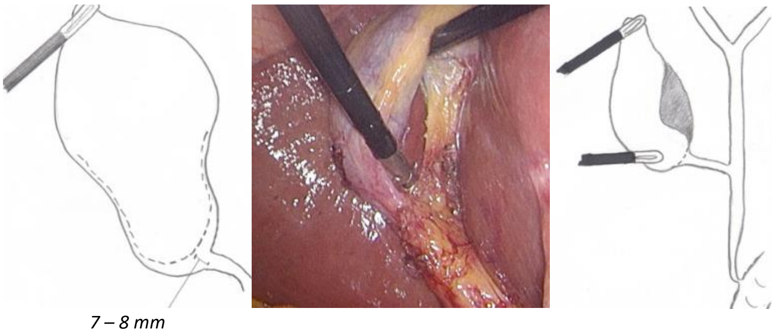

Figure 5. Start of dissection 7-8 mm distal to central margin of infundibulum.

All dissection of the cystic duct and artery starts at the infundibulum

k) The serosa is now mobilized from the initial incision on the infundibulum of the gall bladder. This is done by traction, blunt or sharp dissection, in the direction towards the cystic duct and artery, subsequently exposing these structures for about 1 cm (nearly as taking off pants) (Figure

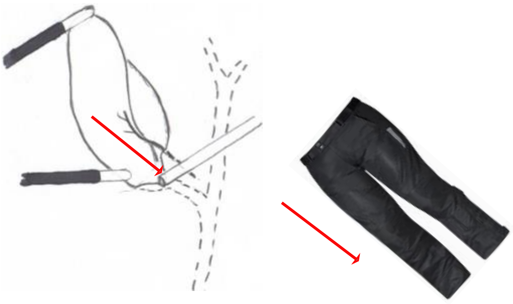

Figure 6. Dissection of the gallbladder hilus.

l) For optimal exposure of the area of cystic duct, it is usually helpful to retract the infundibulum medially and then dissect serosal adhesions and connective tissue off the lateral part of the cystic duct and the central part of the infundibulum from behind. When completed and the infundibulum is retracted laterally to see the hilar region anterior it is usually easier to discriminate the cystic duct and artery (Figure 7).

Figure 7.

a. Showing medial retraction of the infundibulum and dissection from behind the gallbladder
b. Showing lateral retraction of the infundibulum showing hilar region from anterior view.

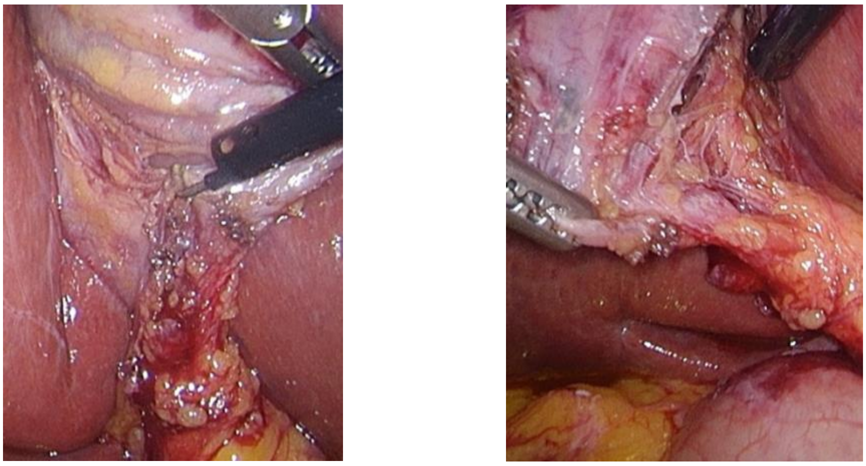

a. Infundibulum retracted medially for dissection on the posterior side.

b. Infundibulum retracted laterally for dissection on the ventral side.

m) The cystic duct and artery are connected by a connective tissue sheeth and may be dissected with the hook, picking up small bites, examined for no ductal structure, and then divided (Figure 8a)

n) The two structures may also be separated with a dissecting instrument with care not to injure the cystic duct, and even more the common bile duct in cases with a very short or missing cystic duct.

a.

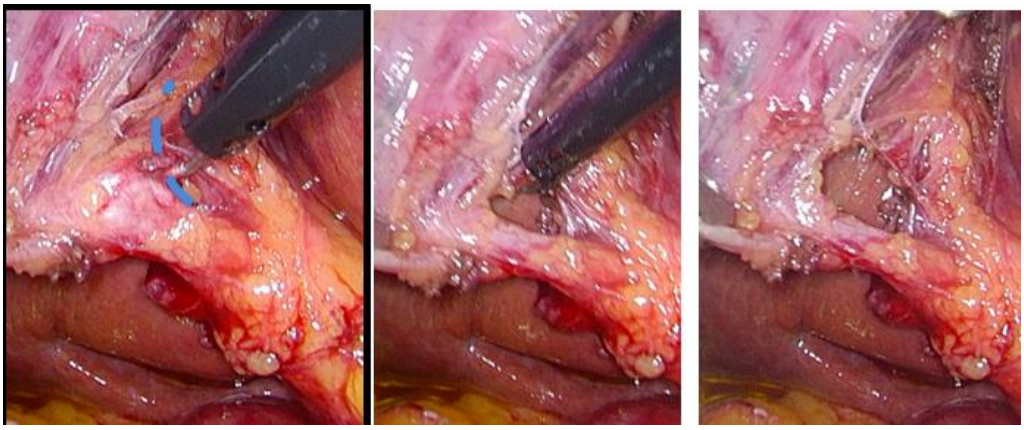

Figure 8. Dissection of hilar structures.

o) All connective tissue and fatty tissue between the cystic duct and artery and the previously established window behind the gall bladder is then dissected. The result is now the window with two structures, the cystic duct and the cystic artery and the anatomy is exposed with a 100% certainty (Figure 9).

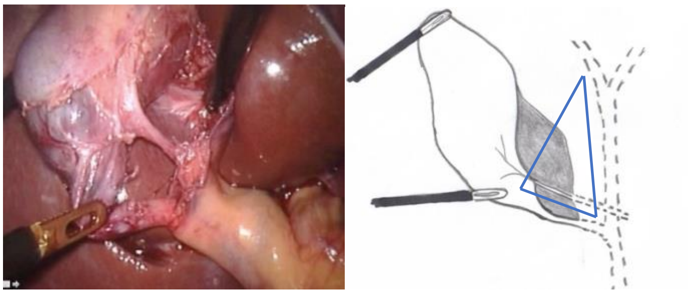

Figure 9. Calots triangle (Cystohepatic triangle) with medial margin being the common hepatic duct, caudal margin being the cystic duct and the lateral margin being the gallbladder

p) The cystic duct may now be closed by clips. The first of the two or three clips that shall remain on the cystic duct stump, shall be the one applied nearest to the common bile duct as the anatomic control is optimal when the first clip is applied.

q) The cystic artery is also clipped with one or two clips remaining on the central stump.

r) The gallbladder is dissected from the liver bed by monopolar diathermy, avoiding perforation, and then delivered in an endobag at the umbilical. Incision may be enlarged to allow passage.

s) Leaked gallstones from a perforation of the gallbladder has to be removed to avoid subsequent abscess formation. Leaked bile should be irrigated and rinsed with saline water.

t) If an intraoperative cholangiography is required, a clip is applied onto the infundibulum. An opening in infundibulum central to the clip or in the very first part of the cystic duct is made with scissors. The cystic duct is examined for stones and a catheter 4 or 5 F is introduced by various means. The patient is tilted to the right not to have the bile ducts projected over the spine. The introduction method may vary.

u) During infusion of contrast fluid imaging interruptedly by a C-arm will show the biliary tree.
