# Supplementary material for: Evaluating the Role of Surgical Telementoring in the Acquisition of Surgical Skills in Laparoscopic Cholecystectomy: Protocol for a Pilot Randomized Controlled Trial
Source: JMIR Res Protoc. 2026 Apr 17;15:e73159. doi: 10.2196/73159 (PMC13089673; doi:10.2196/73159)
Supplement: Checklist 1 [file resprot-v15-e73159-s004.pdf]

SPIRIT 2025 Checklist for SURGTEACH Protocol

Protocol Title: Evaluating the Role of SURGical TElementoring in Acquisition of Surgical Skills of Laparoscopic Cholecystectomy (SURGTEACH). Protocol for a Pilot Randomized Controlled Trial.

Note: Line numbers refer to the Word document, which uses continuous line numbering in the left margin. Section headers and key terms are provided to facilitate locating items.

==
========== ADMINISTRATIVE INFORMATION
==

Item 1 - Title: Identifying the document as a protocol for a randomised trial

Location: Title page, Lines 1-3 Content: "Evaluating the Role of SURGical TElementoring in Acquisition of Surgical Skills of Laparoscopic Cholecystectomy (SURGTEACH). Protocol for a Pilot Randomized Controlled Trial."

Item 2 - Trial registration: Registry name and registration number

Location: - Abstract, Results section (Line ~69): "Trial registration: ClinicalTrial.gov NCT06421584, 14.01.2026" - Results section, Completed activities (Line ~590 -591): Registration confirmation

Item 3a - Protocol version: Date and version identifier

Location: Abstract, Line ~112 Content: "Protocol version 1.0."

Item 3b - Amendments: Description of protocol amendments

Location: Ethics and dissemination section (Lines ~577-579) Content: "Any protocol modifications will be submitted to REK for approval before implementation. Amendments will be documented with date and rationale, communicated to investigators and trial registry, and reported in the final publication."

Item 4a - Contact for public queries

Location: Title page, Corresponding Author (Lines ~16-18) Content: Khayam Butt, MD. Department of Gastrointestinal Surgery. Nordlandssykehuset Bodø. Parkveien 95, 8005 Bodø, Norway. Phone: +47 995 60 985 Email: Khayam.Ashraf.Butt@nordlandssykehuset.no / dr_khayam@hotmail.com

Item 4b - Contact for scientific queries

Location: Same as 4a (Lines ~16-18)

Item 5 - Role and responsibilities of sponsors and funders

Location: Funding Statement section (Lines ~765-771) Content: "This is an investigator-initiated trial. The trial sponsor is Nordland Hospital Trust, represented by the principal investigator (KB). We received a grant from Helgelandssykehuset HF to conduct the trial. Olympus and Proximie provided the cloud solution for the final recruitment phase. Neither company had any role in study design, data collection, data analysis, data interpretation, or manuscript preparation. The investigators maintained full independence in all scientific decisions."

Item 6 - Roles and responsibilities of contributors

Location: Author Contributions section (Lines ~740-744) Content: "Conceptualization: KB, KMA; Methodology: KB, LOT, KMA; Formal analysis: LOT; Investigation: KB, OS; Resources: BE, GH; Writing – Original Draft: KB; Writing – Review & Editing: all authors; Supervision: KMA, BE; Project administration: KB; Funding acquisition: KB, KMA."

== INTRODUCTION ==

Item 7 - Background and rationale

Location: Introduction section (Lines ~77-200) Content covers: - Surgical training challenges and efficiency demands (Lines ~78-81) - Geographical challenges in Northern Norway (Lines ~84-104) - Learning curves in laparoscopic surgery (Lines ~105-113) - Evidence gap and systematic reviews on telementoring (Lines ~114-133) - LapCo TT framework and communication principles (Lines ~134-144) - Assessment methods (GOALS) (Lines ~147-164) - Need for RCTs and pilot trials (Lines ~168-173)

Item 8 - Objectives and hypotheses

Location: Introduction section (Lines ~181-185) Content: "We aim to conduct a pilot RCT to collect data to inform a future full-scale RCT evaluating whether real-time surgical telementoring, combined with structured postoperative coaching, is superior to traditional on-site mentoring in developing laparoscopic cholecystectomy skills among surgical residents. Our primary hypothesis is that residents receiving telementoring will show a 3- to 5-point improvement in GOALS scores compared with the control group."

Secondary objectives (Lines ~186-190): "(1) evaluate the feasibility of recruitment, randomization, and intervention delivery; (2) assess the acceptability of the telementoring system among residents and mentors; (3) identify logistical challenges requiring modification before a definitive RCT; and (4) provide preliminary effect size estimates to refine sample size calculations for a future full-scale trial."

== METHODS: PARTICIPANTS, INTERVENTIONS, AND OUTCOMES

===
==========

Item 9 - Study design

Location: Methods, Study design and setting (Lines ~203-208) Content: “This study is a parallel, 2-group, assessor-blind, multicenter pilot RCT. Participating surgical residents are allocated in a 1:1 ratio to either the intervention group, which receives real-time intraoperative telementoring and postoperative coaching, or the control group, which receives traditional intraoperative mentoring. The intention-to-treat (ITT) principle will be applied. The study adheres to the latest SPIRIT 2025 Statements.”

Item 10a - Setting: Study sites, dates

Location: Methods section (Lines ~210-215) Content: Four hospitals within the Northern Norwegian Health Care Trust initially assessed. Two selected as active sites: Sandnessjøen and Mo i Rana. Timeline in Results section (Lines ~593-601): Recruitment commenced Spring 2024, expected completion May 2026, data analysis November 2026, manuscript submission January 2027.

Item 10b - Eligibility criteria for participants

Location: Eligibility criteria section (Lines ~259-293) Residents (Lines ~260-269): - General surgery residents in years 1 to 6 - More than 10 laparoscopic procedures performed - Completed mandatory courses - Agreement with communication model

Mentors (Lines ~271-277): - Consultant surgeon with >5 years experience - Acquainted with GOALS score assessment - LapCoNor TT course completion required

Patients (Lines ~278-283): - Gallstone disease without clinical history of cholecystitis - BMI < 38 - No previous upper abdominal laparotomy - No previous percutaneous gallbladder drainage - Informed consent provided

Item 10c - Eligibility criteria for study sites

Location: Methods section (Lines ~212-224) Criteria: surgical volume, resident availability, geographic location, telementoring infrastructure, availability of telementors and on-site mentors….

Item 10d - Eligibility criteria for those delivering interventions

Location: Mentors section (Lines ~271-277) Content: Consultant surgeon with >5 years experience, GOALS assessment familiarity, LapCoNor TT course completion.

Item 11a - Description of intervention(s)

Location: Interventions section (Lines ~340-423) Comprehensive description including: - Table 1: Comparison of Intervention and Control Group (Lines ~349) - Pre-procedure setup

and coaching (Lines ~355-362) - Intraoperative telementoring with Lapco “six steps” protocol (Lines ~364-377) - Postoperative coaching using GROW model (Lines ~397-407)

Item 11b - Description of control/comparator(s)

Location: Control Group section (Lines ~408-423) Content: Traditional training with direct in-person mentorship, intraoperative mentoring, and standard informal postoperative feedback.

Item 11c - Criteria for modifying allocated interventions

Location: Methods section (Lines ~289-293) Content: “Residents will continue their standard surgical training during the study period. No restrictions will be imposed on other educational activities (e.g., simulation training and courses).” Also (Lines ~341-343) regarding emergency intervention criteria.

Item 11d - Strategies to improve adherence to interventions

Location: Methods section (Lines ~290-293 and ~346-348) Content: “To promote participant retention, procedures will be scheduled within a 3-day block to minimize scheduling conflicts.” “The mentors and mentees will sign a contract accepting the educational setting and the prerequisites before participating in the trial.”

Item 12 - Primary outcome(s)

Location: Outcomes, Primary outcomes section (Lines ~433-451) Content: GOALS and NOTSS scores. - GOALS: 5 subscales (depth perception, bimanual dexterity, efficiency, tissue handling, autonomy), scored 1-5 each, maximum 25. - Assessment by 3-member expert panel reviewing video footage.

Item 13 - Secondary outcome(s)

Location: Secondary outcome measurements section (Lines ~452-472) - Satisfaction scores: 5-point Likert scale (Lines ~453-458) - Patient-related outcomes: Oslo Classification of Intraoperative Unfavorable Incidents, Clavien-Dindo Classification (Lines ~459-471)

Item 14 - Participant timeline

Location: Participant timeline section (Lines ~318-326) Content: CONSORT flowchart in Figure 5. Residents informed of randomization 1 week before procedures. 5 procedures within 3 days. Data collection timeline in Figure 6 (Line ~502).

== METHODS: ASSIGNMENT OF INTERVENTIONS ==

Item 15 - Allocation sequence

Location: Allocation and blinding section (Lines ~452-467) Content: "Computer-generated stratification will be used for randomization… the randomization process will be stratified by trainees' experience, explicitly distinguishing between those who had performed 5-10 laparoscopic cholecystectomies and those who had performed 10-20."

Item 16 - Allocation concealment

Location: Allocation and blinding section (Lines ~305-309) Content: "The randomization process will use a predetermined statistical sequence generated by an external researcher who was not directly engaged in the study. The allocation sequence will be concealed using a centralized web-based randomization system. Personnel enrolling participants will not have access to the sequence until after enrollment."

Item 17 - Blinding

Location: Blinding section (Lines ~333-339) Content: "Blinding the trainees is not feasible given the characteristics of the intervention. Nevertheless, the evaluators who assessed performance needed to be aware of the assignment of individuals to specific groups… any unintentional unblinding would be documented. No emergency unblinding procedures are required because the intervention poses no direct safety risk."

== METHODS: DATA COLLECTION, MANAGEMENT, AND ANALYSIS ==

Item 18 - Data collection methods

Location: Data collection and management section (Lines ~487-496) Content: Video recordings stored in OR recording system, edited by KB into predefined segments, anonymized and randomized, distributed to evaluation groups. Questback questionnaire for assessments. SurveyMonkey for data collection. Patient data from EHR review. Timeline in Figure 6.

Item 19 - Data management

Location: Data collection section (Lines ~496-500) Content: "The results will be collected via SurveyMonkey© and uploaded to a secure server." Data protection officer approval referenced (Line ~881).

Item 20 - Statistical methods

Location: Statistical Analysis section (Lines ~505-539) Content: - Primary analysis: independent t-test or Mann-Whitney U test (Lines ~505-510) - Effect sizes: Cohen's d (Lines ~508-510) - Secondary analyses: NOTSS comparisons, within-group improvement (Lines ~511-516) - Multivariate analyses with specified covariates (Lines ~517-520) -

Exploratory analyses: learning curves, subgroup analyses (Lines ~525-531) - Feasibility outcomes (Lines ~532-537) - Missing data: available-case analysis (Lines ~556-558)

Item 21 - Data monitoring

Location: Data monitoring section (Lines ~548-562) Content: "Given the pilot nature and low-risk intervention, a formal Data Monitoring Committee was not established. The principal investigator will review safety data after every 5 residents." Quarterly monitoring including recruitment rates, protocol deviations, technical system performance, and adverse events.

Item 22 - Sample size

Location: Sample size section (Lines ~573-486) Content: Based on GOALS SD of 2.5, hypothesized 3-5 point improvement, 0.8 power, $p<0.05$, 20% dropout rate. Full RCT requires 12 residents per group. Pilot includes 5 residents per arm (n=10 total).

== METHODS: ETHICS AND DISSEMINATION ==

Item 23 - Research ethics approval

Location: Ethics and dissemination section (Lines ~564-565) Content: "The study was approved by the ethical committee (REK HELSE NORD 32592) and the data protection officer (DPO) at Nordlandssykehuset Hospital trust, Bodø, Norway."

Item 24 - Protocol amendments

Location: Ethics section (Lines ~577-579) Content: "Any protocol modifications will be submitted to REK for approval before implementation. Amendments will be documented with date and rationale, communicated to investigators and the trial registry, and reported in the final publication."

Item 25 - Consent

Location: Ethics section (Lines ~566-570) Content: "Surgical residents will be informed that their participation is entirely optional, that there will be no repercussions if they choose to withdraw, and that they may withdraw without providing a reason. Upon signing in, all surgical residents must sign an electronic permission document and a mentee-mentor contract (intervention arm)." Patient consent: Lines ~574-576.

Item 26 - Confidentiality

Location: Data collection section (Lines ~491-492) Content: "These standardized, edited records will be anonymized and randomized for each resident." Ethics approval includes data protection officer oversight (Line ~564-565).

Item 27 - Declaration of interests

Location: Conflicts of Interest section (Lines ~763-764) Content: "The authors declare that they have no conflicts of interest."

Item 28 - Harms: Plans for collecting and reporting harms

Location: Secondary outcome measurements section (Lines ~460-472) Content: Intraoperative unfavorable incidents classified using Oslo Classification (Grade 1-3). Clavien-Dindo Classification for postoperative complications.

Item 29 - Post-trial care

Location: Ethics section (Lines ~571-573) Content: "This educational intervention does not pose direct risks to participants beyond those inherent in standard surgical training. No specific post-trial care provisions are required. Residents receive no compensation for participation."

Item 30 - Dissemination policy

Location: Dissemination Plan section (Lines ~717-725) Content: "Results of this pilot study will be submitted for publication in an international peer-reviewed journal, regardless of outcome (positive, negative, or inconclusive). Findings will be presented at relevant surgical education and telemedicine conferences." Specific conferences listed: - Norwegian Surgeons' Annual Meeting, October 2026 - AMEE Conference, August 2026, Vienna - ASiT Annual Conference, 2027

Item 31 - Data sharing

Location: Data Availability Statement section (Lines ~731-739) Content: "The datasets generated during this study will be available from the corresponding author upon reasonable request following publication of the primary results. Anonymized data may be deposited in a suitable repository, subject to ethical approval and data protection regulations. The full study protocol is available on ClinicalTrials.gov. The statistical analysis plan will be finalized before database lock and made available upon request."

== OPEN SCIENCE ==

Item 32 - Open science practices

Location: Data Availability Statement (Lines ~735-737) Content: "The full study protocol is available on ClinicalTrials.gov. The statistical analysis plan will be finalized before database lock and made available upon request. Data collection instruments will be made available as supplementary materials."

==
========== PATIENT AND PUBLIC INVOLVEMENT
==
==========

Item 33 - Patient and public involvement

Location: Patient and Public Involvement section (Lines ~754-762) Content: "This pilot study was designed without formal patient or public involvement in the research design, conduct, or reporting plans. Patients are included solely as surgical cases, with the study focusing on surgical trainee education rather than patient-centred outcomes. Surgical residents, the primary study participants, were consulted informally during protocol development to assess the acceptability and feasibility of the telementoring intervention.

For the planned definitive RCT, we will consider establishing a stakeholder advisory group, including patient representatives and surgical trainees, to inform outcome selection, dissemination strategies, and study conduct."

==
========== APPENDICES
==

Item 34 - Appendices

Location: Appendices section (Lines ~775-780) Content: - Appendix 1: LapCo-Nor involvement in supervision of included mentors - Appendix 2: GROW-model adoption to telementoring sessions - Appendix 3: SPIRIT checklist 2025 - Appendix 4: Detailed description of the step-by-step surgical procedure

This checklist was prepared following the SPIRIT 2025 Statement (Chan A-W, et al. SPIRIT 2025 statement: updated guideline for protocols of randomised trials. Lancet. 2025;405(10491):e19-e27).
